# UNPAID CAREGIVERS’ PROCESS OF COLLABORATING DURING OLDER ADULT HOSPITAL TO HOME TRANSITIONS

**DOI:** 10.1093/geroni/igad104.1390

**Published:** 2023-12-21

**Authors:** Daniel Liebzeit, Olivia Geiger, Saida Jaboob, Samantha Bjornson

**Affiliations:** The University of Iowa, Iowa City, Iowa, United States; The University of Iowa, Iowa City, Iowa, United States; The University of Iowa, Iowa City, Iowa, United States; The University of Iowa, Iowa City, Iowa, United States

## Abstract

Unpaid/family caregivers often provide support critical to older adult hospital to home transitions, but commonly lack time and preparation for their role. There is limited evidence regarding important collaboration and resources for caregivers during the transition. The objective of this grounded theory study was to examine caregivers’ process of collaborating with others during older adult hospital to home transitions. Interviews were conducted with unpaid caregivers of adults aged 60 and older and recently discharged from a medical/surgical unit at a large Midwestern teaching hospital, were audio-recorded, and transcribed verbatim. Data were analyzed using open, axial, and selective coding. Caregiver participants (N = 16) included spouses (n = 8), friends (n = 4), children (n = 3), and siblings (n = 1) of an older adult transitioning from hospital to home, were mostly female (n=14), and all were white, non-Hispanic. A conceptual model was developed which illustrates participants’ process of collaborating with a caregiving team, providers, networks, and other resources to support themselves and their older person. The process began with the participants taking on and identifying their role, and ultimately led to either supporting their older person’s progress towards independence or providing long-term caregiver support. Conditions, including finances, caregiver health, expectations, proximity, and availability, impacted the collaborative process. Consequences varied based on participants’ approach to caregiving, such a team versus individual approach. Implications include opportunities to promote active engagement of caregivers in the transition process and facilitation of a team-based approach to supporting the older person and caregivers.

